# Supplementary material for: Antifouling Lipids from Marine Fungi of the Beibu Gulf
Source: Metabolites. 2025 Nov 5;15(11):721. doi: 10.3390/metabo15110721 (PMC12654144; doi:10.3390/metabo15110721)
Supplement: Supplementary file 1 [file metabolites-15-00721-s001.zip › metabolites-3974948-supplementary.pdf]

Supplementary material

Antifouling Lipids from Marine Fungi of the Beibu Gulf

Mengfan Qi [1,2], Wang Jiang [3], Huaqing Huang [1], Lu Lu [1], Zhiwei Su [3], Xiaowei Luo [1,2], Chenghai Gao [1,2], Yonghong Liu [1,2,*] and Xinya Xu [1,2,*]

[1] Institute of Marine Drugs, Guangxi Key Laboratory of Marine Drugs, Guangxi University of Chinese Medicine, Nanning 530200, China
[2] University Engineering Research Center of High-Efficient Utilization of Marine Traditional Chinese Medicine Resources, Nanning 530200, China
[3] College of Agriculture, Guangxi University, Nanning 530004, China
* Correspondence: yonghongliu@scsio.ac.cn (Y.L.); xuxy@gxtcmu.edu.cn (X.X.)

Table S1. The internal transcribed spacers (ITS) sequence of fungal strains.

No.	Strain number	ITS sequence
1	GXIMD 00527	TGTGACATACCTATACGTTGCCTCGGCGGATCAGCCCGCGCCCCGTAAAACGGGACGGCC<br>CGCCCGAGGACCCCTAAACTCTGTTTTTAGTGGAACTTCTGAGTAAAACAAACAAATAAA<br>TCAAAACTTTCAACAACGGATCTCTTGGTTCTGGCATCGATGAAGAACGCAGCAAAATGC<br>GATAAGTAATGTGAATTGCAGAATTCAGTGAATCATCGAATCTTTGAACGCACATTGCGC<br>CCGCCAGTATTCTGGCGGGCATGCCTGTTCGAGCGTCATTTCAACCCTCAAGCTCAGCTTG<br>GTGTTGGGACTCGCGGTAACCCGCGTTCCCCAAATCGATTGGCGGTCACGTCGAGCTTCC<br>ATAGCGTAGTAATCATACACCTCGTTACTGGTAATCGTCGCGGCCACGCCGTAAAACCCC<br>AACTTCTGAATGTTGACCTCGGATCAGGTAGGAATACCCGCTGAACTTAAGCATATCAAA<br>A
2	GXIMD 00543	TACCTGATCCGAGGTCACCTGAAGAAAAATGGTTGGACGTCGGCTGGCGCCCGGCCGGC<br>CCTAAATCGAGCGGGTGACAAAGCCCCATACGCTCGAGGACCGGACACGGTGCCGCCGC<br>TGCCTTTCGGGCCCGTCCCCCGGGGGGACGACGACCCAACACACAAGCCGGGCTTGAT<br>GGGCAGCAATGACGCTCGGACAGGCATGCCCCCCGGAATGCCAGGGGGCGCAATGTGCG<br>TTCAAAGACTCGATGATTCACTGAATTCTGCAATTCACATTACTTATCGCAGTTCGCTGCG<br>TTCTTCATCGATGCCGGAACCAAGAGATCCATTGTTGAAAGTTTTGACTGATTTTATATTC<br>AGACTCAGACTGCATCACTCTCAGGCATGAAGTTCAGTAGTCCCCGGCGGCTCGCCCCCG<br>AGAGGACTCCCCGCCGAAGCAACAGTGTTAGGTAGTCACGGGTGGGAGGTTGGGCGCCC<br>GGAGGCAGCCCGCACTCAGTAATGATCCTTCCGCAGGTTCACCCTACGGAAG
3	GXIMD 00545	GCGCACTTGTTGTTTCCTGGGCGGGTTCGCTCGCCACCAGGACCACCAAATAAACCTTTTT<br>TATGCAGTTGCAATCAGCGTCAGTACAAACAATGTAAATCATTTACAACTTTCAACAACG<br>GATCTCTTGGTTCTGGCATCGATGAAGAACGCAGCGAAATGCGATACGTAGTGTGAATTG<br>CAGAATTCAGTGAATCATCGAATCTTTGAACGCACATTGCGCCCTTTGGTATTCCAAAGG<br>GCATGCCTGTTCGAGCGTCATTTGTACCCTCAAGCTTTGCTTGGTGTTGGGCGTTTTTGTCT<br>TTGGTCGCCCAAAGACTCGCCTTAAAGTGATTGGCAGCCGGCCTTTCTGGTTTCGCAGCGC<br>AGCACATTTTTGCGCTTGCCATCAGCAAAACGGCAATCCATCAAGCCTCCTTCTCACGTTT<br>GACCTCGGATCA
4	GXIMD 00533	GGTCTACCACCGGGATGTTCATAACCCTTTGTTGTCCGACTCTGTTGCCTCCGGGGCGACC<br>CTGCCTTCGGGCGGGGGCTCCGGGTGGACACTTCAAACTCTTGCGTAACTTTGCAGTCTGA<br>GTAAATTTAATTAATAAATTAAAACTTTTAACAACGGATCTCTTGGTTCTGGCATCGATGA

		AGAACGCAGCGAAATGCGATAAGTAATGTGAATTGCAGAATTCAGTGAATCATCGAATC<br>TTTGAACGCACATTGCGCCCCCTGGTATTCCGGGGGGCATGCCTGTTCGAGCGTCATTTCA<br>CCACTCAAGCCTCGCTTGGTATTGGGCAACGCGGTCCGCCGCGTGCCTCAAATCGACCGG<br>CTGGGTCTTCTGTCCCCTAAGCGTTGTGGAAACTATTCGCTAAAGGGTGCTCGGGAGGCTA<br>CGCCGTAAAACAAACCCATTTCTAAGGTTGACCTCGGATCAGGTAGGGATACCCGCTGAA<br>CTTAAGCATATCAATAAGCGGAGGAA
5	GXIMD 00547	TGCAAATAAATGCGTCGGCGGGCGCCGGCCGGGCCTACGGAGCGGAAGACGAAGCCCC<br>ATACGCTCGAGGACCGGACGCGGTGCCGCCGCTGCCTTTCGGGCCCGTCCCCCGGGAGCC<br>GGGGGACGAGGGCCCAACACACAAGCCGGGCTTGAGGGCAGCAATGACGCTCGGACAG<br>GCATGCCCCCCGGAATACCAGGGGGCGCAATGTGCGTTCAAAGACTCGATGATTCACTG<br>AATTCTGCAATTCACATTAGTTATCGCATTTCGCTGCGTTCTTCATCGATGCCGGAACCAA<br>GAGATCCATTGTTGAAAGTTTTAACTGATTGCAAAGAATCACACTCAGACTGCAAGCTTT<br>CAGAACAGGGTTCATGTTGGGGTCTCCGGCGGGCACGGGCCCGGGGGCGAGTCGCCCCC<br>CGGCGGCCAGCAACGCTGGCGGGCCCGCCGAAGCAACAAGGTACAATAGTCACGGGTG<br>GGGAGGTTGGGCCATAAAGACCCGCACTCGGTAATGATCCTTCCGCAGGTTCACCTACGG<br>AAACCTTGTTACGA
6	GXIMD 00548	AACCTCCCACCCGTGACTACTGTACCACTGTTGCTTCGGCGGGCCCGCCAGCGTCCGCTG<br>GCCGCCGGGGGGCTTCTGCCCCCGGGCCCGTGCCCGCCGGAGACCCCAACACGAACACT<br>GTTTCTGAAAGCCTGTATGAATCCGATTCTTTGTAATCAGTTAAAACTTTCAACAATGGAT<br>CTCTTGGTTCCGGCATCGATGAAGAACGCAGCGAAATGCGATAACTAATGTGAATTGCAG<br>AATTCAGTGAATCATCGAGTCTTTGAACGCACATTGCGCCCCCTGGTATTCCGGGGGGCA<br>TGCCTGTCCGAGCGTCATTACTGCCCTCAAGCCCGGCTTGTATTGGGTCCTCGTCCCCCTC<br>CCCGGGGGACGGGCCCGAAAGGCAGCGGCGGCACCGCGTCCGGTCCTCGAGCGTATGGG<br>GCTTTGTCACCCGCTCTGTAGGCCCGGCCGGCGCCAGCCCACGCAACACCTTTTTTTTTCA<br>GGTTGACCTCGGATCAGGTAGGGATACCCGCTGAACTTAAGCATATCAATA
7	GXIMD 00519	GTCACCTGAAGAAAATGGTTGGACGTCGGCTGGCGCCCGGCCGGCCCTAAATCGAGCGG<br>GTGACAAAGCCCCATACGCTCGAGGACCGGACACGGTGCCGCCGCTGCCTTTCGGGCCC<br>GTCCCCCGGGGGGGACGACGACCCAACACACAAGCCGGGCTTGATGGGCAGCAATGAC<br>GCTCGGACAGGCATGCCCCCCGGAATGCCAGGGGGCGCAATGTGCGTTCAAAGACTCGA<br>TGATTCACTGAATTCTGCAATTCACATTACTTATCGCAGTTCGCTGCGTTCTTCATCGATGC<br>CGGAACCAAGAGATCCATTGTTGAAAGTTTTGACTGATTTTATATTCAGACTCAGACTGCA<br>TCACTCTCAGGCATGAAGTTCAGTAGTCCCCGGCGGCTCGCCCCCGAGAGGGCTCCCCGC<br>CGAAGCAACAGTGTTAGGTAGTCACGGGTGGGAGGTTGGGCGCCCGGAGGCAGCCCGCA<br>CTCAGTAATGATCCTTCCGCAGGTTCACCCTACGGA
8	GXIMD 00544	TCTGGGTCCACCTCCCACCCGTGTCTATCGTACCTTGTTGCTTCGGCGGGCCCGCCGTTTCG<br>ACGGCCGCCGGGGAGGCCTTGCGCCCCCGGGCCCGCGCCCGCCGAAGACCCCAACATGA<br>ACGCTGTTCTGAAAGTATGCAGTCTGAGTTGATTATCGTAATCAGTTAAAACTTTCAACAA<br>CGGATCTCTTGGTTCCGGCATCGATGAAGAACGCAGCGAAATGCGATAAGTAATGTGAAT<br>TGCAGAATTCAGTGAATCATCGAGTCTTTGAACGCACATTGCGCCCCCTGGTATTCCGGG<br>GGGCATGCCTGTCCGAGCGTCATTGCTGCCCTCAAGCACGGCTTGTGTGTTGGGCCCCCGT<br>CCCCCTCTCCCGGGGGACGGGCCCGAAAGGCAGCGGCGGCACCGCGTCCGGTCCTCGAG<br>CGTATGGGGCTTTGTCACCTGCTCTGTAGGCCCGGCCGGCGCCAGCCGACACCCAACTTT<br>ATTTTTCTAAGGTTGACCTCGGATCAGGTAGGGATACCCGCTGAACTTAAGCATATCAAT<br>A
9	GXIMD 00541	GTGACATACCTATACGTTGCCTCGGCGGATCAGCCCGCGCCCCGTAAAACGGGACGGCCC<br>GCCCGAGGACCCCTAAACTCTGTTTTTAGTGGAACTTCTGAGTAAAACAAACAAATAAAT

10	GXIMD 00502	CAAAACTTTCAACAACGGATCTCTTGGTTCTGGCATCGATGAAGAACGCAGCAAAATGCG<br>ATAAGTAATGTGAATTGCAGAATTCAGTGAATCATCGAATCTTTGAACGCACATTGCGCC<br>CGCCAGTATTCTGGCGGGCATGCCTGTTCGAGCGTCATTTCAACCCTCAAGCTCAGCTTGG<br>TGTTGGGACTCGCGGTAACCCGCGTTCCCCAAATCGATTGGCGGTCACGTCGAGCTTCCAT<br>AGCGTAGTAATCATACACCTCGTTACTGGTAATCGTCGCGGCCACGCCGTAAAACCCCAA<br>CTTCTGAATGTTGACCTCGGATCAGGTAGGAATACCCGCTGAACTTAAGCATATCAATAA<br>GTCACCTGGAAAAAATGGTTGGAAAACGTCGGCAGGCGCCGGCCAATCCTACAGAGCAT<br>GTGACAAAGCCCCATACGCTCGAGGATCGGACGCGGTGCCGCCGCTGCCTTTCGGCCCCG<br>TCCCCCCGGAGAGGGGGACGGCGACCCAACACACAAGCCGGGCTTGAGGGCAGCAATG<br>ACGCTCGGACAGGCATGCCCCCCGGAATACCAGGGGGCGCAATGTGCGTTCAAAGACTC<br>GATGATTCACTGAATTCTGCAATTCACATTAGTTATCGCATTTCGCTGCGTTCTTCATCGAT<br>GCCGGAACCAAGAGATCCATTGTTGAAAGTTTTAACTGATTGCATTCAATCAACTCAGAC<br>TGCACGCTTTCAGACAGTGTTCGTGTTGGGGTCTCCGGCGGGCACGGCCCGGGGGGCAA<br>AGGCGCCCCCCCGGCGGCCGACAAGCGGCGGCCCGCCGAAGCAACAGGGTATAATAG<br>ACACGGATGGGAGGTTGGGCCCAAAGGACCCGCACTCGGTAATGATCCTTCCGCAGGTC

Table S2. Biofouling settlement on PVC panels of selected lipid fractions during the 90-day marine field trial.

	Time (day)				
lipids	15	30	45	60	90
GXIMD00 543	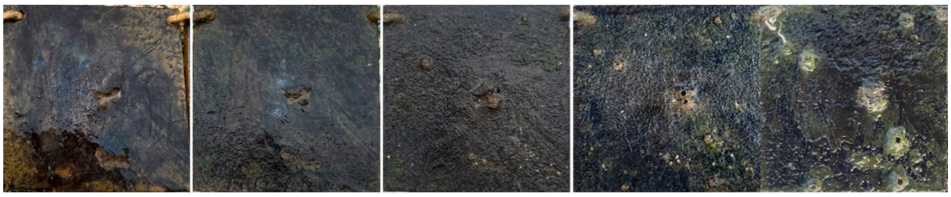				
GXIMD00 541	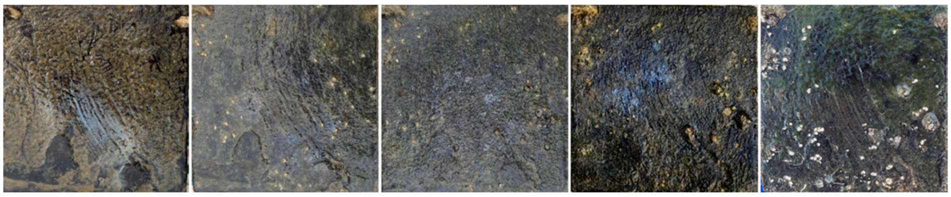				
GXIMD00 533	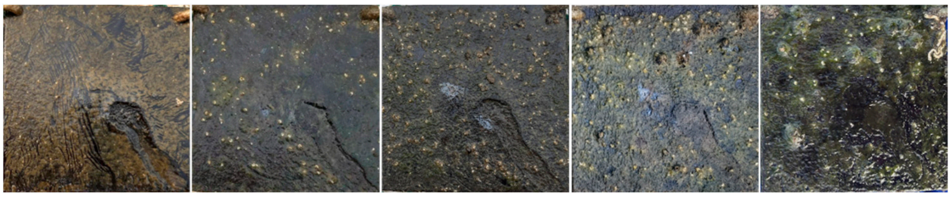				
GXIMD00 527	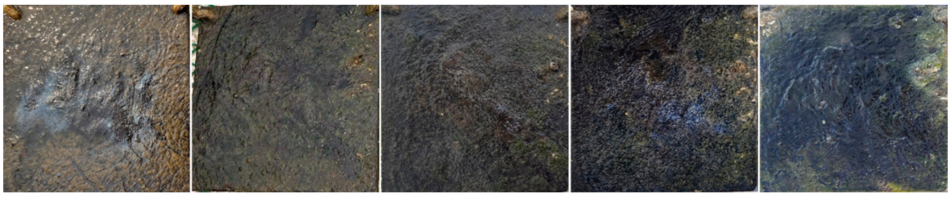				
GXIMD00 548	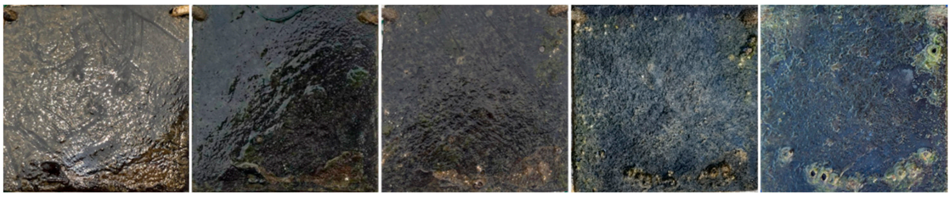				

GXIMD00 519	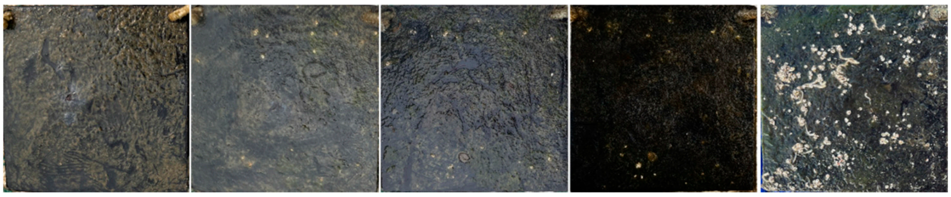
GXIMD00 547	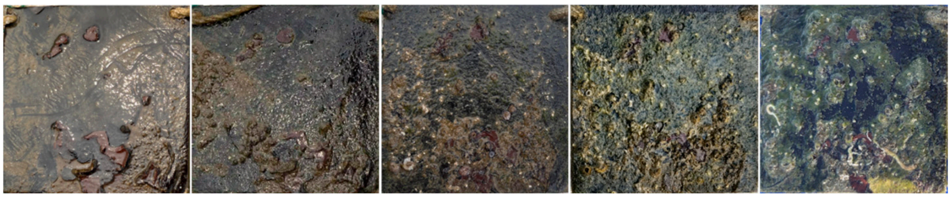
Blank control	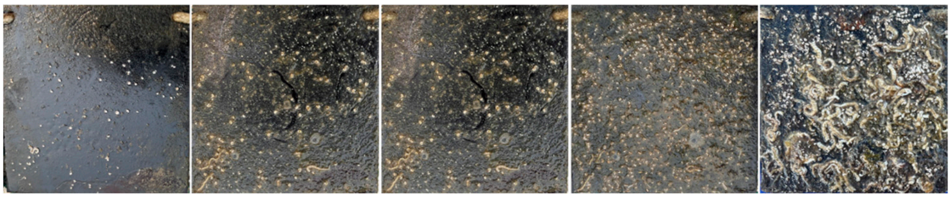
Positive control	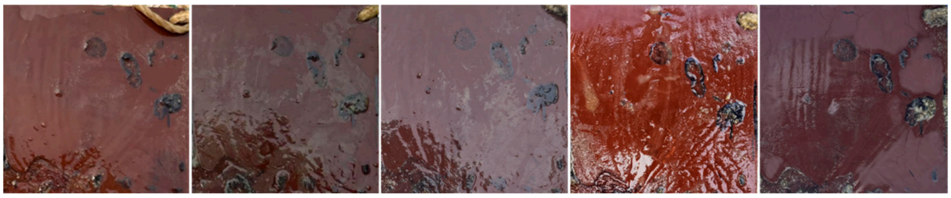
